# Event-related potential and lexical decision task in dyslexic adults: Lexical and lateralization effects

**DOI:** 10.3389/fpsyg.2022.852219

**Published:** 2022-11-09

**Authors:** Patrícia Botelho Silva, Darlene Godoy Oliveira, Amanda Douat Cardoso, Paulo Guirro Laurence, Paulo Sérgio Boggio, Elizeu Coutinho Macedo

**Affiliations:** Social and Cognitive Neuroscience Laboratory, Developmental Disorders Program, Center for Health and Biological Sciences, Mackenzie Presbyterian University, São Paulo, Brazil

**Keywords:** developmental dyslexia, cognitive profile, electroencephalography, potentials related to events, hemispheric lateralization

## Abstract

Developmental dyslexia is a specific learning disorder that presents cognitive and neurobiological impairments related to different patterns of brain activation throughout development, continuing in adulthood. Lexical decision tasks, together with electroencephalography (EEG) measures that have great temporal precision, allow the capture of cognitive processes during the task, and can assist in the understanding of altered brain activation processes in adult dyslexics. High-density EEG allows the use of temporal analyses through event-related potentials (ERPs). The aim of this study was to compare and measure the pattern of ERPs in adults with developmental dyslexia and good readers, and to characterize and compare reading patterns between groups. Twenty university adults diagnosed with developmental dyslexia and 23 healthy adult readers paired with dyslexics participated in the study. The groups were assessed in tests of intelligence, phonological awareness, reading, and writing, as well as through the lexical decision test (LDT). During LDT, ERPs were recorded using a 128-channel EEG device. The ERPs P100 occipital, N170 occipito-temporal, N400 centro-parietal, and LPC centro-parietal were analyzed. The results showed a different cognitive profile between the groups in the reading, phonological awareness, and writing tests but not in the intelligence test. In addition, the brain activation pattern of the ERPs was different between the groups in terms of hemispheric lateralization, with higher amplitude of N170 in the dyslexia group in the right hemisphere and opposite pattern in the control group and specificities in relation to the items of the LDT, as the N400 were more negative in the Dyslexia group for words, while in the control group, this ERP was more pronounced in the pseudowords. These results are important for understanding different brain patterns in developmental dyslexia and can better guide future interventions according to the changes found in the profile.

## Introduction

Developmental dyslexia (DD) is a neurobiological disorder that brings challenges to the person for decoding words, resulting in poor spelling and reading fluency skills. These difficulties typically result from a deficit in the phonological, visual, auditory, and orthographic components of language, being the use of written language deficient, which is often unexpected relative to other cognitive skills and adequate classroom teaching instruction (Lyon, 2003; Schumacher et al., 2007; Handler and Fierson, 2011; American Psychiatric Association [APA], 2014; Nergård-Nilssen and Hulme, 2014). In this sense, we can consider DD as a spectrum of a variety of cognitive and neurobiological changes that reflect behavioral deficits (Carioti et al., 2021).

According to the literature, DD manifests itself differently throughout development and across languages and orthographies (Borleffs et al., 2019; Carioti et al., 2021). Orthographic consistency may be an important factor in the manifestation of symptoms and cognitive profile of DD, also as cross-linguistic aspects (Reis et al., 2020). Differences in orthographies can be attributed to spelling depth as well as syllabic complexity and these aspects directly influence the acquisition and development of reading skills. Word reading is a more complex task for deep orthographies than for superficial ones and, as a result, when dyslexics become adults there is a reduction in the gap when compared to normal readers. Although, this pattern is not a sign of better reader performance itself, but can be explained by the orthographic transparency in written languages such as Spanish, Italian, and Portuguese for example (Carioti et al., 2021).

Although the diagnosis occurs predominantly in childhood, cognitive, and neurobiological patterns remain in adulthood. In children, difficulties affect word decoding skills as well as losses are present in establishing the relationship between spelling patterns and word pronunciation (Handler and Fierson, 2011; Snowling and Hulme, 2012). Symptoms in adult life are presented differently due to the occurrence of compensatory behaviors and strategies developed to minimize reading and academic or non-academic impairments (Schelke et al., 2017). Therefore, compensatory mechanisms are developed throughout the life-span to reduce functional impairment such as greater activation of the left superior temporal region and inferior parietal region in working memory tasks, as well as greater activation of the left inferior frontal gyrus in phonological discrimination tasks (Nergård-Nilssen and Hulme, 2014; Soriano-Ferrer and Piedra Martínez, 2017; Mahé et al., 2018). However, neurobiological and cognitive markers of dyslexia remain over their lifetime (Schelke et al., 2017).

Although difficulties in cognitive skills such as working memory, phonological awareness, and rapid automatic naming are also present in adulthood, difficulties in reading and writing skills are the main cognitive markers (Soriano-Ferrer and Piedra Martínez, 2017; Carioti et al., 2021). One of the main indicators of adult dyslexia is spelling and letter problems stemming from a lack of orthographic knowledge. As long regular readers benefit from successful synchronization between different cerebral systems related to visual, auditory, and semantic processes during reading, dyslexics present asynchrony between the visual and auditory systems in the brain, termed the asynchrony phenomenon (Breznitz, 2002; Nergård-Nilssen and Hulme, 2014). At the same time, the meta-analysis from Soriano-Ferrer and Piedra Martínez (2017) about neurobiological basis of Dyslexia confirms the absence of hemispheric lateralization in dyslexic children and adults during written language tasks.

Lexical decision tasks provide cognitive assessments of the processes underlying word recognition skills. The task paradigm allows the evaluation of lexical access accuracy and speed, as well as the lexicon development level (Balota and Chumbley, 1984; Berberyan et al., 2021). Lexical decision tasks help to verify the orthographic-semantic (word) and phonological (pseudowords) processing in word recognition (Shaul, 2013). In lexical decision tasks, brain activity is different for words, and pseudowords, taking into account that performance differences can also be explained by differences in decision making (Shaul et al., 2012; Shaul, 2013; Berberyan et al., 2021). Also, differences in lexical decisions tasks. This differentiated pattern of word-related brain activity is not present in dyslexic adults, reflecting the orthographic and phonological deficits present in this learning disability (Shaul et al., 2012; Shaul, 2013).

In this context, research on lexical decision tasks with the recording of high-density EEG measures can help us to understand the neurophysiological processes underlying reading providing information before the appearance of a behavioral response. Measurements of neuronal activity using high-density electroencephalography (EEG) have been used in studies with people with dyslexia because they have good temporal accuracy, enabling inferences about cognitive processes during the lexical decision task (Ozernov-Palchik and Gaab, 2016; Perera et al., 2018a).

High-density EEG allows the use of temporal analysis through event-related potentials (ERPs). ERPs are characterized by being sensitive to cognitive parameters triggered by a specific task (Caylak, 2009), and these tasks may be reading tasks. In general, some studies that recorded ERPs in dyslexic children and adults have found, when compared to normal readers data, changes in latency and amplitude in potentials related to visual processing (Kast et al., 2010; Dujardin et al., 2011), orthographic (Taroyan and Nicolson, 2009; Waldie et al., 2012), semantics (Horowitz-Kraus and Breznitz, 2008; Hasko et al., 2013), and cognitive (Taroyan and Nicolson, 2009; Shaul et al., 2012) that are involved in word recognition. Word recognition involves the ability to see a word and recognize its pronunciation effortlessly and instantly. To develop automaticity in word recognition, instructions in phonological awareness, decoding with good skills and knowledge of spelling rules and grapheme-phoneme conversion are required, and finally visual recognition of words (Murray, 2016). Furthermore, differentiated brain activity patterns in dyslexic adults were also observed using EEG measurements (Perera et al., 2018b).

Some ERPs are relevant in word reading and lexical decision analysis in DD. The ERP differences found in dyslexic adults may be related to sub-efficient neural mechanisms (Mahé et al., 2018). Studies have shown that dyslexics present linguistic processing of different patterns in early components (P100, P200, N100, and N2), associated with sensory-perceptual processing and the physical characteristics of stimuli (Taroyan and Nicolson, 2009; Dujardin et al., 2011; Mahé et al., 2018). In dyslexic adults, lower latencies and lower left hemisphere activation of this ERP were reported, as well as greater right hemisphere activation for pseudo object visualization (Mayseless and Breznitz, 2011).

In particular, early components P100 and N170 can also be related to the processing of orthographic structure and letter position in the word, which is important for the recognition of the visual form of word characteristics (Araújo et al., 2015). Some authors (Carreiras et al., 2014; Coch and Meade, 2016; Mahé et al., 2018) report N170 greater amplitudes in normal readers during the reading process of real words, as well as phonological sensitivity and how the sounds of letters form words, showing a lexicality effect. The greater N170 amplitude in response to words and spelling sensitivities is not present in dyslexic adults, and this ERP difference can be a hallmark of the neurobiological profile of DD (Mahé et al., 2013; Carreiras et al., 2014). Furthermore, N2, ERP measured between 135 and 205 ms can also be related to lexical access and showed lower amplitudes in adult dyslexics compared to good readers (Mahé et al., 2018). In addition to possible group differences, it is important to report hemispheric effects concerning the component called N1–measured between 150 and 180 ms (Araújo et al., 2015), present with greater amplitude in the left hemisphere of both dyslexics and good readers.

Regarding later processing during word reading, the N400 is a linguistic component that is sensitive to semantic manipulation (Kutas and Federmeier, 2011). Furthermore, other linguistic manipulations of items, such as word frequency, can modify this component activity (Kutas and Federmeier, 2011). During word recognition processing, both words, pseudowords, or stimuli with sound or orthographic irregularity, reflect changes in N400. This ERP is sensitive to factors prior to recognition stages such as orthographic neighborhood, frequency, and orthographic and phonological similarity, and may be associated with memory and retrieval of linguistic information (Kutas and Federmeier, 2011). Changes in the N400 component reflect compensatory changes in adult dyslexics, whose semantic aspects are processed through morphology as a way to compensate for phonological impairments (Cavalli et al., 2017).

Another component that may be altered in DD is P600, which is described as related to syntactic violation (grammar-imposed restrictions) (Kutas and Federmeier, 2011). This is observed in both the visual and auditory stimuli. van Herten et al. (2006) suggest that the P600 also reflects the engagement of executive and cognitive processes in error monitoring and reprocessing services to resolve the uncertainty of responses during linguistic processing, analyzing, and reanalyzing processes already carried out.

Considering the importance of spelling regularity and the distinct cognitive profiles found in cross-linguistic studies, the present research aims to elucidate the electrophysiological bases underlying cognitive processing and hemispheric activity triggered by a lexical decision task in dyslexic Brazilian adults. The aim of this study was to compare and measure the ERP patterns of adults with DD, Brazilian Portuguese readers, and control readers, as well as to compare reading patterns between the groups to verify possible orthographic influences on the cognitive profile of the groups. From this, the study can contribute to a better understanding of EEG and the cognitive basis of dyslexia can present itself in Brazilian Portuguese.

## Materials and methods

### Participants

A total of 20 university adults diagnosed with developmental dyslexia (DG-Adults) and 23 good readers paired with dyslexics (CG-Adults) participated in this study. For the diagnosis of the group of adults with dyslexia, neuropsychological assessments were performed at the Laboratory of Cognitive and Social Neuroscience in which the group participants had a cognitive profile compatible with DD. To ensure homogeneity among the participants, the pairing was performed according to age, gender, and education level. Thus, participants’ ages ranged from 18 to 41 years (*M* = 24.97 + 4.73; *p* = 0.603). Of the 43 participants, 56.5% were women (DG-Adults = 13, CG-Adults = 13; *p* = 0.532), and all had undergraduate courses ongoing or finished. All participants were assessed using a broad battery of neuropsychological, reading, and writing tests to meet the inclusion and exclusion criteria. Inclusion criteria: (1) level of intelligence assessed by the Wechsler Adult Intelligence Scale at or above average (above the 25th percentile) and (2) delay in reading and writing skills in relation to subjects with the same education level for the group of participants with dyslexia and reading and writing skills in the middle range or higher for the control group. Exclusion criteria: Participants with comorbid psychiatric, neurological, truancy, or vision problems were excluded.

### Instruments

Wechsler Adult Intelligence Scale assesses intellectual ability through measures of verbal IQ, performance IQ, and global IQ. In addition, it assesses four cognitive domains that underlie intellectual skills: verbal comprehension, working memory, perceptual organization, and processing speed (Wechsler, 2004).

Word Reading Competence Test 2 (WRCT-2): is composed of 80 items, which are formed by pairs that involve the auditory and visual presentation of a word, which may or may not be congruent. The pairs can be congruent, in which case the spoken word and written word are identical, or incongruent, in accordance with specific types of errors in the written words. The incongruent pairs are of four types: written words with visual changes of letter position in the word, letter omission, word with phonological changes, and words with visual confusion of letters (de Oliveira et al., 2009; de Oliveira, 2014; Oliveira et al., 2014; Dias et al., 2016).

Word Dictation Writing Test for adults (WDWTA): The test consists of 50 items in which it seeks to assess the ability to spell irregular words that depend on the use of proper spelling rules, which are dictated by the subject (de Oliveira, 2014).

Phonological awareness test 2 (PAT-2): The PAT-2 is a test adapted to the adult population and has the same items as the children’s version (Capovilla et al., 2011). The test consists of 64 items divided into subtests of rhyme, alliteration, syllabic addition, syllabic subtraction, phonemic addition, phonemic subtraction, syllabic transposition, phonemic transposition, and pun. Each item has a semantic distractor, phonological distractor, inverse-rule distractor, and unspecific distractor (de Oliveira et al., 2008; de Oliveira, 2014; Oliveira et al., 2014).

**FIGURE 1:**
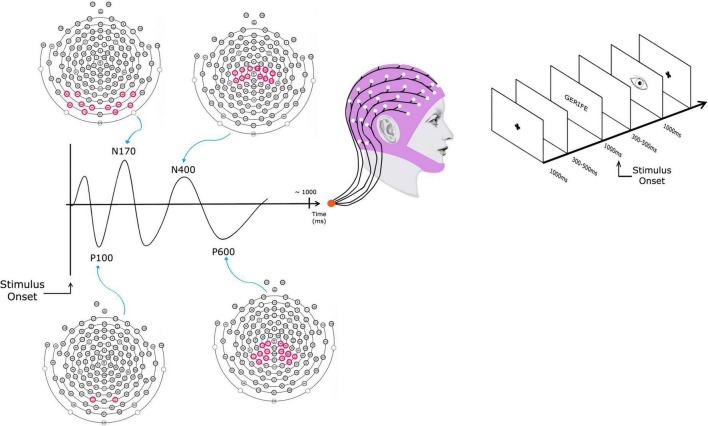
A schematic presenting the event-related potential (ERP) to lexical decision task stimulus obtained by averaging the electroencephalography (EEG) signal of multiple stimulus presentations. The data was collected with a 128-channel array of scalp electrodes from which the EEG was recorded. The electrodes chosen for the four components were: P100 (Occipital), N170 (Occipital-Temporal), N400 (Central), and P600 (Central-Parietal). In the electrode’s figures, the purple circles indicate which electrodes were used for each component.

Lexical Decision Task: The lexical decision task was created considering the feasibility criteria for the application and recording of electrophysiological responses in the high-density electroencephalogram. The test consists of 540 items, consisting of 180 regular words, 180 pseudowords, and 180 quasi-words. The stimuli were selected according to psycholinguistic properties of length and frequency in the Portuguese language. The syllabic structure of the stimuli was counterbalanced between the structures CVCVCV, VCVCV, CCVCVCV, and VCCVCV. In addition, the number of letters of the stimuli varied between 5 and 7 letters, so there was no influence of the variable length during the processing of the items. All words have a medium or high frequency in Portuguese according to the Corpus NILC Universidade de São Carlos.^1^ Regarding regularity, regular and rule words were selected. The quasi-words are divided into three categories: 60 pseudohomophones, 60 visual exchanges, and 60 phonological exchanges. Such categorization is based on the study by Proverbio and Adorni (2008). The pseudowords were constructed from sequences of decodable letters and syllables, but not derived from real words. Considering this factor, the frequency values of bigrams of the task stimuli with five and six letters were measured, according to data from Justi and Justi (2009). The results showed that the pseudowords present bigrams with very low frequency in the Portuguese language (mean of 19.64, SD = 1.37). The test stimuli were prepared in bitmap format files (BMP) with a resolution of 800 × 600 pixels. The font used was Courier New, black, bold type, size 18 on a white background. Each stimulus appears on the screen for 2 s, after they disappear, the subject must press a button if he judges the word as real or invented, followed by a screen with a picture of an eye so that the subject can blink. After the response is emitted, a blank screen with a central cross appears for 3 s for the subsequent presentation of the next stimulus. The lexical decision task items were presented in six blocks of 90 items each, and the items in the categories were randomized along the blocks. The number of correct answers, omissions errors (i.e., not pressing the button), and reaction time. Response times of incorrect responses and those shorter than 100 msec or longer than 2.5 SD above the subject’s mean were eliminated. Figure 1 shows the sequence of presentation of the lexical decision task screens on the right.

### Equipment

128-channel Electroencephalography Device, Electrical Geodesics, Inc., Eugene, OR, USA model EEG System 300. The equipment is composed of an amplifier model Net Amps 300, transformer with isolation, articulated arm to support the amplifier, license for acquisition, and data analysis software Net station, six Geodesic hydrocele model electrode networks, Macintosh CPU for data acquisition, 23″ monitor for data monitoring, software for calculating the sources generating the signals (GeoSource Estimation Software), package for Event Related Evoked Potential (PST, Inc., Savannah, GA, USA), E-prime workstation to couple to EEG (*Net Station*), computer desktop Dell, hardware for the experiments, 17″ Liquid Crystal Display (LCD) monitor with video *splitter and switch*, b response for Electrical Geodesics, Inc (EGI), *single clock*, Audio Visual (AV) device.

### Procedures

The study was conducted in accordance with the requirements of the ethics committee of Mackenzie Presbyterian University, after evaluation and approval of the research project (CEP/UPM no. 1305/12/2010). The participants were contacted and informed of the research objectives. After reading and signing the letter of information and the term of consent, neuropsychological and computerized reading and writing assessments were performed. Evoked potentials were recorded using a Geodesic EEG System 300. Regarding the collected EEG tracing, a pre-processing phase was initially carried out, which contained: (a) 0.1 Hz filter (*High Pass Filtering*), (b) 30 Hz (*Low Pass Filtering*), (c) segmentation of the trace considering the 200 ms prior to the presentation of the screen containing the proposal and the decision and the 1,200 ms after, (d) artifact detection was performed before averaging to discard epochs in which eye movements, blinks, excessive muscle potentials, or amplifier blocking occurred. Artifacts were considered as channels with a Max-Min variation greater than 200 μV (with a time window of 640 ms). Those with more than 20% of artifacts were considered as bad channels and were automatically rejected. In addition, bad segments were considered those with more than 10 bad channels or blinking.

After the pre-processing phase, the post-processing phase followed, which included: (a) replacement of bad channels with interpolated values based on neighboring channels, (b) average of the potentials obtained in the segmentation considering the factors described above (a data file was created containing all evoked potentials incorporating data from all participants), and (c) correction for the baseline, which is the tracing obtained in portions of 200 ms prior to the presentation of stimuli. The data were then subjected to statistical processing considering the mean amplitude for the occipital P100, occipitotemporal N170, parietal N400, and center-parietal late positive complex (LPC) potentials as dependent variables. The occipital P100 was analyzed in the temporal window between 30 and 150 milliseconds and the selected electrodes were #70 and 83. The occipitotemporal N170 component was analyzed in the temporal window of 140–270 milliseconds. Left hemisphere electrodes n° 64, 65, 68, 69, 73, and 74 were selected, as well as the right hemisphere electrodes n° 82, 88, 89, 90, 94, and 95. The potential N400 of the central-parietal region was analyzed in the temporal window between 300 and 500 ms. Electrodes no. 7, 30, 31, 36, 37, and 42 were selected in the left hemisphere, as well as the following right hemisphere electrodes: 80, 87, 93, 104, 105, and 106. Finally, the center-parietal P600 component was analyzed in the temporal window between 450 and 850 ms. Electrodes # 60, 61, 53, 54, 37, 31, 42, and 52 were selected for the analysis of the left hemisphere, and electrodes # 80, 87, 93, 79, 86, 92, and 85 were selected for LPC analysis in the right hemisphere. The choice of electrode sites and time windows for measuring and quantifying ERP components of interest was chosen based on previous literature that showed that the components usually reach their maximum over these areas (Proverbio and Adorni, 2008; Dujardin et al., 2011). Figure 1 presents the electrodes that were analyzed for each component.

### Data analysis

To understand if the control and dyslexia groups differed in relation to their cognitive profile, we conducted several Student’s *t*-tests comparing both groups for the WAIS-III, WDWTA, WRCT-2, and PAT-2 measures. We also compared the performance of both groups for the behavioral data in the lexical decision task. Both groups were compared in terms of accuracy, reaction time, and missions. We also calculated Cohen’s d to analyze the effect size.

To compare the electrophysiological responses of both groups in the lexical decision task, we carried out several repeated measures ANOVAs. For the within factor, we used the hemisphere of the brain and the lexicality of the words in the task. For the between-factor analysis, we used the dyslexic-control group. We also calculated the generalized eta squared (η^2^) to analyze the effect size of the findings. For all analyses, we considered a significant *p*-value of <0.05.

## Results

The performance of each group was evaluated using the WAIS-III, WDWTA, WRCT-2, and PAT-2. We compared the two groups to understand the differences in the profiles of each group. Their performance and the differences between the groups are shown in Table 1. No differences were found in the WAIS-III and PAT-2 measures, but significant differences were observed in WDWTA-2 and WRCT-2 (*p* = 0.033). A large effect between both groups was found in all WDWTA and WRCT-2 measures, with the exception of the time in the WDWTA, which was marginally large.

**TABLE 1:** Mean and standard deviation (SD) for the control and dyslexia group and their comparison.

Tests	Measures	Control mean (SD)	Dyslexia mean (SD)	*t*	*P*-value	Cohen’s d
WAIS-III	Total IQ	120.80 (11.17)	119.20 (11.65)	0.31	0.758	0.14
	Verbal IQ	115.40 (10.46)	109.10 (19.50)	0.90	0.383	0.40
	Executive IQ	123.60 (8.97)	115.60 (23.55)	1.00	0.336	0.57
	Verbal comprehension	116.10 (8.89)	102.90 (26.49)	1.49	0.163	0.67
	Perceptual organization	122.20 (8.74)	109.40 (27.46)	1.40	0.188	0.63
	Working memory	112.30 (15.40)	95.80 (29.07)	1.59	0.136	0.71
WDWTA	Total number of correct answers	43.30 (3.83)	31.60 (10.02)	3.45	**0.005**	1.54
	Time (in ms)	5567.15 (1499.21)	7310.33 (2727.86)	1.77	0.098	0.79
WRCT-2	Total number of correct answers	72.40 (4.77)	61.60 (5.72)	4.59	**<0.001**	2.05
	Time (in ms)	1130.73 (432.45)	1819.39 (807.94)	2.38	**0.033**	1.06
PAT-2	Total number of correct answers	54.2 (7.54)	49.7 (7.99)	1.30	0.211	0.58
	Time (in ms)	15096.38 (1436.93)	17959.32 (5032.82)	1.73	0.113	0.77

WDWTA, Word Dictation Writing Test for adults; WRCT-2, Word Reading Competence Test for adults; PAT-2, phonological awareness test 2. Bold values are significant results.

### Lexical decision task: Behavioral data

**FIGURE 2:**
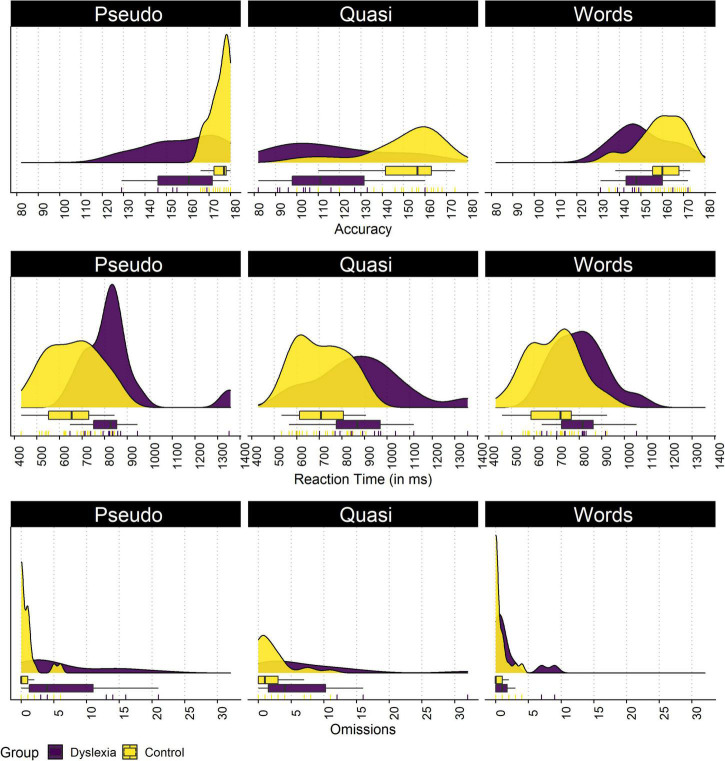
Distributions of the control and dyslexia group for the total number of correct answers, the reaction time, and the total number of omissions in the lexical decision task. The density plots present the density estimate of each variable, the boxplot present the median and quartiles, and the rugs present each data point individually.

In the lexical decision task, we compared both groups for each measure. The plot of each group dispersion in the lexical decision task is shown in Figure 2. Significant differences were found in the number of correct answers in words (*p* = 0.045, *d* = 0.76), pseudowords (*p* = 0.002, *d* = 1.56), and quasi words (*p* < 0.001, *d* = 1.51). For reaction time, significant differences of large magnitude were present in words (*p* = 0.006, *d* = 1.02), pseudowords (*p* = 0.002, *d* = 1.33), and quasi words (*p* = 0.009, *d* = 1.13). Regarding omissions, significant differences of large magnitude were found in pseudowords (*p* = 0.009, *d* = 1.28) and quasi words (*p* = 0.046, *d* = 0.89), and no differences were found for regular words (*p* = 0.123, *d* = 0.66).

### Lexical decision task: Electroencephalography data

We evaluated four different components of the EEG measures during the lexical decision task and analyzed the effects of lexicality, group, and hemisphere. We also observed an interaction between these factors. The effects and interactions are listed in Table 2.

**TABLE 2:** The effects and interactions for the P100, N170, N400, and P600 components of the electroencephalography during the lexical decision task, their *p*-values, and their effect size.

Effects and interactions	P100		N170		N400		P600	
	*p*	η^2^	*p*	η^2^	*p*	η^2^	*p*	η^2^
Lexicality	0.502	0.001	0.591	<0.001	**0.029**	**0.007**	**0.019**	**0.009**
Group	0.632	0.004	0.564	0.007	0.601	0.006	0.061	0.085
Hemisphere	**0.038**	**0.005**	**0.010**	**0.034**	**0.013**	**0.020**	**<0.001**	**0.023**
Lexicality × group	0.162	0.005	0.368	0.001	0.473	0.002	0.311	0.003
Hemisphere × group	0.421	0.003	0.067	0.017	0.054	0.012	0.607	<0.001
Hemisphere × lexicality	0.571	<0.001	0.340	0.002	0.269	0.003	**<0.001**	**0.005**
Hemisphere × lexicality × group	0.804	<0.001	0.238	0.002	0.176	0.004	0.952	<0.001

Bold values are significant results.

**FIGURE 3:**
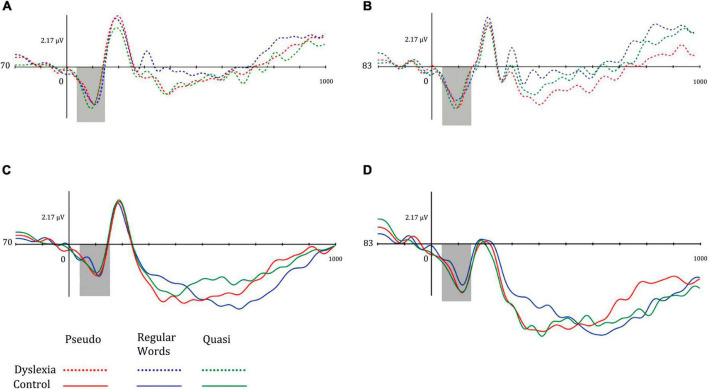
Mean amplitude of the P100 component for **(A)** dyslexia group and left hemisphere, **(B)** dyslexia group and right hemisphere, **(C)** control group and left hemisphere, and **(D)** control group and right hemisphere. Dotted lines represent the dyslexia group, while solid lines represent the control group. Red-colored lines represent pseudo words, blue-colored lines represent regular words, and green-colored lines represent Quasi words.

The P100 amplitude means were higher in the right hemisphere in both groups. The means of P100 in the DG were greater for the pseudowords in both hemispheres, while in the CG, the mean of the left P100 was greater in the pseudowords and in the right P100 for the quasi-words. Figure 3 shows the P100 components of the different groups.

**FIGURE 4:**
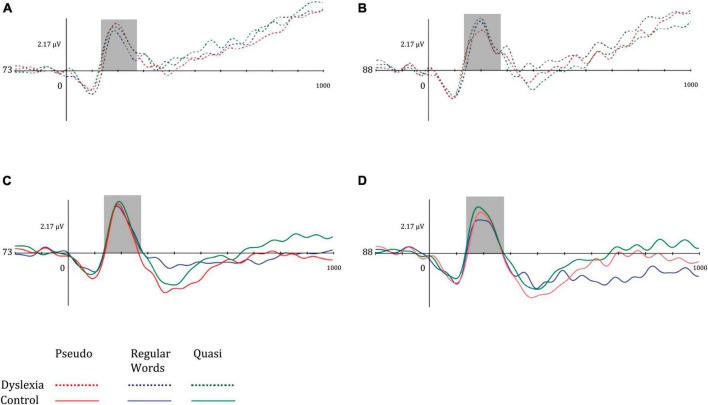
Mean amplitude of the left occipitotemporal N170 component for **(A)** dyslexia group and left hemisphere, **(B)** dyslexia group and right hemisphere, **(C)** control group and left hemisphere, and **(D)** control group and right hemisphere. Dotted lines represent the dyslexia group, while solid lines represent the control group. Red-colored lines represent pseudo words, blue-colored lines represent regular words, and green-colored lines represent Quasi words.

Regarding the N170 component data, the mean amplitudes of the N170 were more negative in the control group and in the left hemisphere. It is noted, in general, that the mean N170 in the dyslexia group was higher in the right hemisphere, while the opposite pattern was observed in the control group. Regarding lexicality, the amplitude means were higher for words, followed by quasi-words and pseudo-words. Figure 4 illustrates the mean amplitudes of the N170 in both hemispheres for both groups.

**FIGURE 5:**
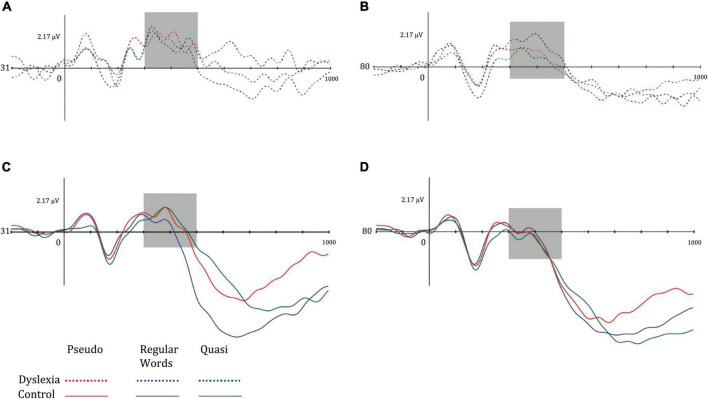
Mean amplitude of the left mid-parietal N400 component for **(A)** dyslexia group and left hemisphere, **(B)** dyslexia group and right hemisphere, **(C)** control group and left hemisphere, and **(D)** control group and right hemisphere. Dotted lines represent the dyslexia group, while solid lines represent the control group. Red-colored lines represent pseudo words, blue-colored lines represent regular words, and green-colored lines represent Quasi words.

Regarding the N400 component, the mean amplitudes of the centro-parietal N400 in the dyslexia and control groups were greater in the left hemisphere. Amplitudes were more negative in the Dyslexia group for words, while in the control group, the N400 was more pronounced in the pseudowords. Furthermore, the mean amplitude of this potential was greater in the left hemisphere. The marginal interaction effect between hemisphere and group indicated that in the dyslexic group, the N400 amplitudes were smaller in the left hemisphere. Figure 5 illustrates the mean N400 amplitudes in the left and right hemisphere of the dyslexic and control groups in the different lexical classes of the lexical decision task.

**FIGURE 6:**
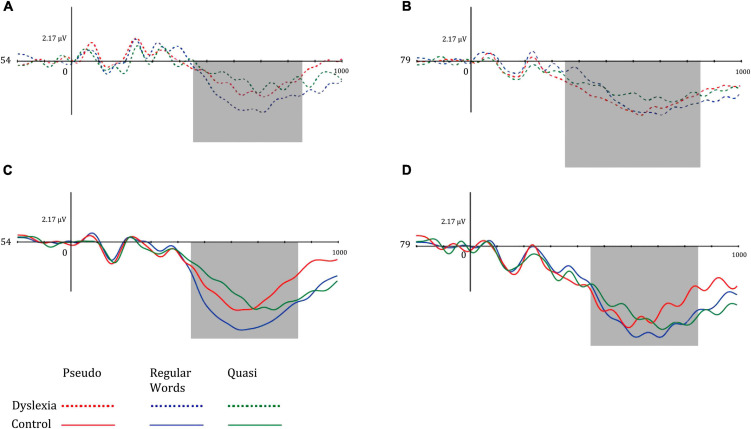
Mean amplitude of the left mid-parietal P600 component for **(A)** dyslexia group and left hemisphere, **(B)** dyslexia group and right hemisphere, **(C)** control group and left hemisphere, and **(D)** control group and right hemisphere. Dotted lines represent the dyslexia group, while solid lines represent the control group. Red-colored lines represent pseudo words, blue-colored lines represent regular words, and green-colored lines represent Quasi words.

Regarding the P600 component, the mean amplitude values in the center-parietal region were lower in the dyslexic group and higher in the left hemisphere in both groups. The P600 amplitude was greater in the left hemisphere, and the dyslexic group had reduced amplitudes. Regarding lexicality, the P600 was more pronounced for words and higher than for pseudowords and quasi-words. Figure 6 illustrates the mean P600 amplitudes in the left hemisphere (electrode 54) and right hemisphere (electrode 79) of the dyslexic group (above) and control (below) in the different lexical classes of the lexical decision task.

## Discussion

The aim of this study was to compare and measure the ERP patterns of adults with DD, Brazilian Portuguese readers, and control readers, as well as to compare reading patterns between the groups to verify possible orthographic influences on the cognitive profile of the groups. Regarding behavioral data, dyslexics did not presented deficits in phonological awareness in PAT-2 and WAIS verbal and working memory measures. Although some studies describe these changes in the profile of dyslexics in relation to good readers (Nergård-Nilssen and Hulme, 2014; van Setten et al., 2016), in adulthood these changes are more variable and seem to depend on orthographic regularity (Carioti et al., 2021). The fact that we did not find disadvantages in dyslexics can be explained by the orthographic transparency of Portuguese. According to Reis et al. (2020), dyslexia symptoms are less marked in clear orthographies and phonological awareness appears to be less of a problem in adulthood. On the other hand, significant losses were found in reading, and writing skills, both in accuracy and speed. Deficits in reading and writing are the most important cognitive markers in the cognitive profile of dyslexic adults in transparent orthographic systems (Carioti et al., 2021). The dyslexic group presented low performance in accuracy on the reading task by recognizing words and pseudowords–WRCT-2, which was also verified in van Setten et al. (2016), Paz-Alonso et al. (2018), and Ozernov-Palchik et al. (2021). The writing data followed the same pattern from the reading ones, since the WDWTA results from the dyslexic group were significantly lower than the normal readers and writing impairments keep being a cognitive feature in the profile of dyslexic adults (Shaul, 2012; Nergård-Nilssen and Hulme, 2014).

Regarding the results of the lexical decision task, the same pattern of difficulties of dyslexic readers in relation to good readers for the measures of accuracy and speed of linguistic processing was observed, as well as a greater number of omission items. These results corroborate the findings of other studies on lexical decision tasks (Bergmann and Wimmer, 2008; Mahé et al., 2012). The results showed a longer reaction time and a higher omission rate for quasi words for both groups of readers in the present study. In the study by Mahé et al. (2012), both typical and dyslexic readers had longer reaction times and higher numbers of errors in pseudowords derived from real words, and this pattern was even higher in the dyslexic group. Taroyan and Nicolson (2009), Shaul et al. (2012), and Shaul (2013) also found similar behavioral results. These results may indicate word identification processes related to orthographic familiarity of stimuli, in which proximity to real words makes stimulus analysis difficult (Taroyan and Nicolson, 2009; Mahé et al., 2012).

Regarding the electrophysiological data, the P100 component presented a similar amplitude pattern in both groups, with larger amplitudes in the right hemisphere. The P100 component has been associated with visuospatial attention and in the detection of physical properties of stimuli, that can present alteration in dyslexic readers (Dujardin et al., 2011). The present study did not find group differences concerning the amplitude of this component, however, comparing the average amplitudes in relation to the type of stimuli, high amplitudes of P100 can be observed in the DG for pseudowords in both hemispheres, while for CG the same component was higher in the right hemisphere for pseudowords and quasi words. Dujardin et al. (2011) found smaller amplitudes of P100 in the occipitotemporal area in the left hemisphere during pseudoword processing in dyslexics. In the present study, this pattern was observed in dyslexics, which may indicate changes in early brain activity related to non-specialization in the beginning processing of the visual form of words.

Regarding N170, the present study found a hemispheric effect, with more negative amplitudes in the CG and the left hemisphere. In addition, the mean N170 in the DG group was higher in the right hemisphere during quasi-word processing. This brain activation profile is seen in functional neuroimaging studies in which dyslexics do not show hemispheric specialization, but a right-lateralized activation pattern (Soriano-Ferrer and Piedra Martínez, 2017). In processing linguistic stimuli such as word reading, the right hemisphere is activated and ends up being inhibited by the left hemisphere when the word meaning is found (Shaul et al., 2012). This pattern did not appear to occur in the dyslexic group.

In the present study, the mean amplitude of this component was greater in words, followed by quasi-words and pseudowords, thus showing that the closer the proximity to the appropriate orthographic pattern, the greater the amplitude of the N170 component. This component is related to the initial recognition of linguistic stimuli, as well as differentiates orthographic stimuli from symbols and is related to the lexicality of the stimuli (Casaca, 2017). Even if we didn’t find a lexicality effect, we can check that N170 is related with sensitive to spelling familiarity (Coch and Mitra, 2010), and involves lexical access (smaller range for unfamiliar words), in addition to indicating parallel processing between the visual recognition of words and access to their lexical representations (Araújo et al., 2015). The study by Casaca (2017) with adults who are good readers and speakers of Portuguese also found only a marginal effect in relation to the N170 component and lexicality effect. Therefore, this effect may be related to the processing of linguistic information from Portuguese, since for good readers the effect is not found either.

From this, dyslexics process orthographic information less efficiently and more slowly, with reduced activity in the left occipitotemporal region, which is responsible for orthographic identification and phonological integration. Thus, this information is not properly accessed (Savill and Thierry, 2011). Thus, this impairment of information processing and integration, which results in changes in the activity of the N170 as shown in this study, as well as difficulties in the judgment of words as found in the lexical decision test (LDT). From this, dyslexics present impairments in visual and phonological integration, as well as in lexical processing. These results also corroborate what was found by Mahé et al. (2012), who showed that dyslexics did not show a hemisphere effect in relation to the type of stimulus, as was observed in the control group.

Korinth et al. (2012), when analyzing the relationship of the N170 in readers with and without reading fluency problems, found that the ERP was higher for linguistic stimuli in the left hemisphere. Furthermore, there was a positive correlation between N170 and reading speed, as more fluent readers had a greater amplitude of N170 for linguistic stimuli. According to the authors, impairments in the analysis of physical structures of stimuli prevent further lexical processing, corroborating the findings of the present study, since dyslexics did not present the hemispheric specialization of N170 and seemed to process linguistic stimuli as distinct categories in the right hemisphere as a compensation mechanism.

Sensory deficits in perception and discrimination of the phonological characteristics of stimuli are observed in different languages, being these deficits considered universal (Goswami et al., 2011). Studies with different languages have shown changes in the N170 component (for review, see Premeti et al., 2022), so this deficit may be independent of linguistic regularity.

Data from N400 showed a hemispheric effect (*p* = 0,013) with greater amplitudes in left hemisphere in both groups. In the present study, we also found a general lexicality effect (*p* = 0.029). Amplitude means from dyslexics were more negative in words, whereas in the controls, it was more pronounced in pseudowords. Studies with lexical decision tasks show that N400 is more negative for pseudowords than words, showing a lexicality effect, with an inverse effect on word frequency (Coch and Mitra, 2010; Berberyan et al., 2021). This effect occurs since the pseudowords do not have a lexical representation and therefore the lexical identification process requires additional effort, with the N400 being the initial process of the decision process and responsible for memory retrieval (Berberyan et al., 2021). This effect was observed only for the CG of the present study, and this result may show the semantic changes in word processing during the lexical decision. Shaul et al. (2012) also found a differentiated pattern of activation in relation to words and pseudowords derived from real words.

The N400 component is concerned with grapheme-phoneme conversion and lexical access, as well as lexical-semantic interpretation. Studies in different languages show mixed results regarding differences between dyslexic and good readers. The inconsistency in the results of this component may be related to several factors such as the task, type of stimulus, age of the participants, or reading difficulty (Premeti et al., 2022). Furthermore, since the N400 component is related to grapheme-phoneme and lexical-semantic and orthographic conversion processes, linguistic regularity can influence the mechanisms of linguistic information processing by dyslexic readers and good readers. The study by Lima (2008) discusses the importance of grapheme-phoneme conversion for an intermediate orthography such as Portuguese. The study sought to analyze the effect of grapheme-phoneme conversion on phonological processing from the effect of word and pseudoword stimulus extension. The results showed that this effect occurs depending on the task, reading aloud or lexical decision, and that in intermediate orthographies the modulation of extension effects is more apparent for Portuguese since grapheme-phoneme correspondence is used in tasks that the task presents phonological salience, however, it is not the exclusive strategy of phonological processing, being the lexical access also relevant in the processing of words. Thus, the activation of the N400 component is related to the relevant cognitive processes during the reading of words in Portuguese, which involves both grapheme-phoneme conversion and direct lexical access. Thus, different languages and linguistic regularities have different grapheme-phoneme conversion rules and, therefore, learning and reading difficulties are not the same (Carioti et al., 2021). Thus, the results of the present study may indicate that for Brazilian Portuguese, the processes of lexical access and grapheme-phoneme conversion shown in the behavioral results also reflect difficulties with brain functioning.

As seen in the N400 amplitude pattern, the P600 results also indicate a lexicality effect (*p* = 0.019) with greater amplitude for words, corroborating the literature (Berberyan et al., 2021). The greater amplitude for words is associated with word frequency and the semantic integration that occurs during lexical decision tasks (Berberyan et al., 2021). Grammatical violations cause semantic consequences that interfere with syntactic aspects of the language, which causes amplitude changes in N400, as well as changes in P600 due to the effect of sensitivity to syntactic changes (Kutas and Federmeier, 2011). The P600 results indicated greater amplitudes in the left hemisphere in both groups (*p* < 0.001) and smaller amplitudes in dyslexics, contrary to the results found by Cavalli et al. (2017), who found greater amplitudes in dyslexics and discussed these data considering compensatory mechanisms resulting from impairments in early word processing. Thus, dyslexics in the present study seem to require more time to process stimuli than in the study mentioned above, since they cannot process stimuli in the late time window. In addition, the DG showed longer reaction time and omission in behavioral data, indicating that they needed more time to process linguistic stimuli. readers access visual and auditory channels in parallel and process information from perceptual channels to linguistics in the left hemisphere. Dyslexics, on the other hand, do not have the characteristics of the words stored in the lexicon, and need to access visual patterns and later the auditory ones, with the visual information arriving late to auditory processing and the information being processed in the right hemisphere. Information processing in dyslexics is based on early processing in the right hemisphere (Shaul, 2013).

As the P600 is involved in the processing of items with linguistic incongruity or is related to the re-analysis of information (Savill and Thierry, 2011), it seems that the dyslexics in the present study are processing linguistic information still in a late time window (pseudowords with greater linguistic inconsistencies), while the controls re-evaluate the previously decided answer (greater for words). Thus, dyslexics do not show automaticity in reading words, and consequently have lower P600 amplitudes, as well as higher omission and reaction time rates. In addition, P600 has smaller amplitudes under task conditions that have greater uncertainty in stimulus assessment and categorization. Thus, in word recognition tasks, more familiar words may be more easily recognized and have greater P600 amplitudes (van Hees et al., 2017). Thus, when decisions are taken with absolute certainty, the P600 presents greater amplitude, which strengthens the above hypothesis and reaffirms uncertainty in the decision-making process of dyslexics when faced with linguistic stimuli.

From the results described above, cognitive and linguistic processing during decision tasks is altered in adult dyslexics both concerning behavioral and neurophysiological data. The linguistic regularity of Portuguese seems to have a distinct effect on neurophysiological processing and ERPs. However, the literature is scarce regarding the discussion regarding ERPs and different activation patterns compared in different orthographies. New studies need to be conducted with this objective. This study was not carried out without any limitations. One of them is the sample size. Since the total sample consists of 43 participants, this study can be low-powered. Thus, the findings presented in this study should be taken cautiously. Future studies should look into similar effects with a bigger sample to confirm them. A second limitation is that although no significant difference was found between the groups in the WAIS-III, Cohen’s d was high, indicating a possible confounding effect.

## Conclusion

It is concluded that there is a reorganization of brain activity in dyslexic adults, with a predominance of the right hemisphere and little differentiation between lexical categories and recognizable or unrecognizable linguistic stimuli. In addition, dyslexics have much slower word processing than good readers, in addition to having significant impairments since the beginning of written language processing in the brain. The linguistic regularity of Portuguese may have influenced the neurophysiological processing of reading, and further studies are needed seeking to identify the influence of orthography to differentiate readers and the distinct brain processing from that.

## Data availability statement

The data supporting the conclusions of this article will be made available by the authors upon request, without undue reservation.

## Ethics statement

The studies involving human participants were reviewed and approved by the Mackenzie Presbyterian University Ethics Committee. The patients/participants provided their written informed consent to participate in this study.

## Author contributions

PS and DO designed the study, collected the data, analyzed the data, and wrote the manuscript. AC wrote the manuscript. PL and PB analyzed the data and wrote the manuscript. EM designed the study, analyzed the data, and wrote and revised the manuscript. All authors contributed to the article and approved the submitted version.

## Footnotes

1
http://www.linguateca.pt/ACDC/
